# Medicinal and Crop Protection Chemistry: Breaking Barriers, Building Synergies

**DOI:** 10.1002/cmdc.70441

**Published:** 2026-08-26

**Authors:** Giulia Cazzaniga, Giusy L. Fratello, Alexander T. Apostle, Roberto Orru, David M. Barber, Silvia Gazzola

**Affiliations:** ^1^ Science and High Technology Department Insubria University Como Italy; ^2^ Research and Development Weed Control Crop Science Division Bayer AG Frankfurt am Main Germany

**Keywords:** crop protection chemistry, drug discovery, dsRNA, ligand targeted drug conjugate, PROTAC

## Abstract

Recognizing the strong interconnection between human, animal, and environmental health, the “One Health” perspective listed by WHO promotes cross‐disciplinary integration as a key strategy to address global challenges. Within this framework, this perspective article summarizes the key themes and future directions emerging from the conference “Medicinal and Crop Protection Chemistry: Breaking Barriers, Building Synergies,” held in Como, Italy, in October 2025. The meeting highlighted the strong conceptual and technological convergence between medicinal and crop protection science, two disciplines that share common goals in the discovery and development of bioactive compounds despite traditionally evolving as separate sectors. Emphasis was particularly placed on providing an updated overview of the latest advances in drug and agrodrug development through those strategies discussed during the congress, which ranged from established approaches in bioactive molecule discovery, such as bioisosterism and covalent ligand design, to emerging modalities including AI‐assisted discovery and targeted protein degradation technologies. We believe that the congress represented an important opportunity to catalyze dialog across both sectors, encouraging the exchange and adaptation of innovative strategies rather than their simple transfer, with the hope that this interdisciplinary initiative will continue to grow and stimulate future collaborations toward sustainable solutions for global health and food security.

## Graphical abstract

A unified view of medicinal and crop protection chemistry reveals shared scientific progress, common challenges, and new opportunities for cross‐disciplinary collaboration. Bridging expertise across both fields could accelerate innovation and actively encourage more sustainable solutions.

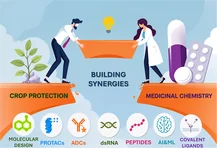

## 1 Introduction

The discovery and development of bioactive compounds can be considered one of the most powerful drivers of human civilization [1].

As organic and medicinal chemists, we often associate the development of bioactive compounds with human medicine, a field that naturally attracts public attention due to its direct and visible impact on people's lives. Medical advances are therefore widely perceived as essential and frequently featured in public discourse. Yet, the same fundamental principles of molecular design and synthesis of novel bioactive molecules are also applied beyond the field of human health, particularly in crop protection. The term crop protection refers to the strategies and active ingredients used to mitigate crop yield losses caused by pests such as weeds, insects, and fungal pathogens. Although essential for ensuring food in a world of continuously growing population [2, 3], crop protection has historically received less attention and investment than its human health counterpart, and its importance remains often poorly visible outside scientific and agriculture context [4, 5].

This lack of attention is particularly concerning today, as the increased spread of resistant species, a phenomenon that is empowered by climate change, is leading to catastrophic control failures through the complete loss of valuable tools. To address this issue, several action committees, such as the Herbicide Resistance Action Committee (HRAC), Fungicide Resistance Action Committee (FRAC), and Insecticide Resistance Action Committee (IRAC), have been established to monitor and develop strategies to globally fight the agrodrug resistance, which now affects thousands of insects, weeds, and fungal species and represents one of the major threats to global food security [6, 7, 8].

However, given the scale and complexity of these phenomena, it is increasingly evident that resistance cannot be effectively addressed by focusing exclusively on agriculture. Similar challenges are threatening human and animal health, where the rapid spread of drug resistance is progressively eroding the efficacy of available therapies and reducing the number of effective treatment options. As an example, Antimicrobial Drug Resistance (AMR), known as a “silent pandemic,” is considered one of the top ten global public health threats to humanity in the 21st century [9]. In parallel, climate change is contributing to the expansion and redistribution of parasitic diseases by altering the geographical range and life cycles of pathogens and their vectors, thereby increasing the risk of transmission in previously unaffected regions and further complicating disease control strategies [10]. In all these sectors, the continuous loss of active compounds highlights the urgent need for integrated approaches capable of combining expertise from different scientific disciplines, including, but not limited to, medicinal chemistry, crop protection, veterinary sciences, biology, and environmental sciences. More specifically, the successful development and sustainable application of a bioactive molecule requires a full understanding of its entire life cycle, spanning from molecular design and chemical optimization to pharmacological and toxicological characterization, biological evaluation, and the investigation of resistance mechanisms. Beyond these aspects, a broader understanding of compound behavior across biological and environmental systems (i.e., their fate, transport, transformation, and persistence) is essential to generate the knowledge required to accurately assess efficacy, safety, and broader impacts under real‐world application conditions. To promote this broader vision, the World Health Organization has increasingly advocated the “One Health” perspective, a framework that recognizes the strong interconnection between human, animal, and environmental health and encourages cross‐disciplinary integration of knowledge to develop more efficient and sustainable strategies [11].

Within this scenario, the first edition of the conference “Medicinal and Crop Protection Chemistry: Breaking Barriers, Building Synergies” (MCPS), held in Como, Italy, on October 16–17, 2025, aimed to promote dialog across these two domains by bringing together experts from academia and industry to explore converging themes in medicinal chemistry and crop protection discovery.

Herein, we aim to summarize and critically discuss the major themes that emerged during the congress, ranging from established chemical strategies for the design and synthesis of novel bioactive compounds, such as scaffold hopping and bioisosterism, to the discovery of new modes or sites of action, as well as the development of covalent ligands.

The second part of this perspective will be dedicated to those strategies discussed during the congress that have been mostly emerging over the last two decades within the field of novel bioactive entity research and development. Indeed, recent trends indicate a clear shift from traditional small, Rule‐of‐Five‐compliant molecules toward emerging modalities [12], which include RNA interference (RNAi), peptide‐based agents, and biologically derived products such as microbial‐ or plant‐based compounds. In parallel, approaches such as targeted protein degradation (e.g., proteolysis‐targeting chimeras [PROTACs]) and drug conjugates are expanding the scope of targetable biological pathways [13], while artificial intelligence (AI) techniques are enabling huge steps to accelerate innovation.

The MCPS s congress and this perspective arose from the organizers' conviction that examining medicinal and crop protection chemistry through a unified lens, could highlight scientific advances, shared challenges, and opportunities for future cross‐disciplinary collaboration, which would accelerate innovation, thereby contributing to a healthier and more sustainable future.

## 2 Classical Approaches for the Development of Novel Bioactive Compounds in Medicinal Chemistry and Crop Protection

### 2.1 Scaffold Hopping and Bioisosterism

**FIGURE 1:**
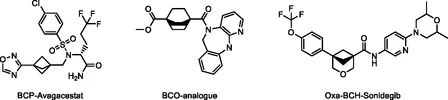
Selected examples of compounds containing bioisosteres of benzene.

Small‐molecule entities have been the cornerstone of the pharmaceutical and crop protection industries since their emergence. In recent decades, new innovations in the pharmaceutical field have extensively shifted this industry away from the traditional Rule‐of‐Five‐compliant bioactive compounds (see also Section 3) [14, 15], although they still remain a viable and valuable modality in the fight against diseases. In comparison, while innovative crop protection strategies, such as biological control agents and genome editing technologies, are attracting increasing investments [16, 17, 18, 19], small molecules continue to represent the predominant class of crop protection products and are expected to maintain a central role for the foreseeable future. It is therefore of utmost importance to further build on tried‐and‐tested strategies that yield results in the optimization of small‐molecule lead candidates. Among others, scaffold hopping ad bioisosterism are those strategies that have afforded much success [20, 21]. Although these strategies have been prominent for a number of decades, they continue to advance, typically via the identification of new chemistry that allows more facile access to molecular architectures of ever‐increasing complexity. In turn, this enables easier fine tuning of compound properties such as target selectivity, breaking resistance, overcoming adverse toxicological effects, suboptimal ADME profiles, as well as being a useful tool to generate novel intellectual property [22]. While there are a variety of approaches to scaffold hopping and bioisosterism, one area of intense focus in recent years has been on the replacement of planar benzene moieties with sp^3^‐rich motifs, often in an effort to overcome the poor “drug‐like” properties typically associated with benzene rings [23, 24, 25]. The most commonly seen small‐ring structures utilized for this endeavor include bicyclo [1.1.1]pentane (BCP), bicyclo [2.1.1]hexane (BCH), bicyclo [3.1.1]heptane (BCHep), bicyclo [2.2.2]octane (BCO), and cubane, with numerous examples being found in the literature where these entities have been successfully deployed (Figure 1) [26, 27, 28].

**FIGURE 2:**
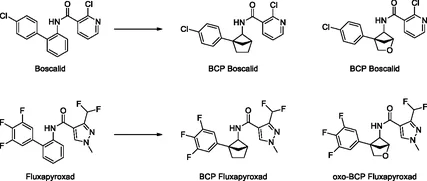
Introduction of BCH and oxa‐BCH bioisosteres into the structures of the SDH‐inhibiting fungicides boscalid and fluxapyroxad.

In comparison to the pharmaceutical industry, in crop protection field the concept of utilizing these complexity‐increasing bioisosteres remains in its infancy, although several examples have recently been reported, and the number is steadily increasing. Mykhailiuk and coworkers used the benzene ring found in the succinate dehydrogenase (SDH)‐inhibiting fungicides boscalid and fluxapyroxad as a template to demonstrate how the BCH motif can serve as a bioisosteric replacement (Figure 2) [29]. The corresponding products were evaluated to compare their properties with the commercial active ingredients, with changes in phosphate‐buffer solubility and metabolic stability in human liver microsomes being the most notable. Mykhailiuk and coworkers also followed up their initial publication with an additional report of a method to access derivatives of several SDH inhibiting compounds bearing different BCH isomers [30].

**FIGURE 3:**
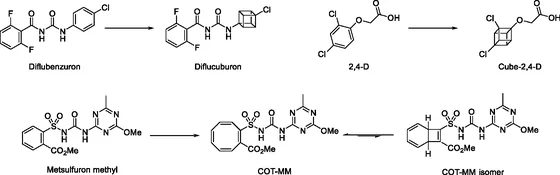
Use of cubane as a bioisostere for the replacement of benzene rings in diflubenzuron, 2,4‐D, and the incorporation of a COT ring into metsulfuron methyl.

Williams and co‐workers have disclosed several interesting reports of using cubane as a bioisosteric replacement for benzene rings. For example, the insecticide diflubenzuron was converted to a compound termed diflucuburon after replacement of a benzene ring with a cubane [31] and, more recently for the replacement of a 2,4‐dichloro‐substituted benzene ring in the herbicide 2,4‐D, affording Cube‐2,4‐D (Figure 3) [32]. This has also been supplemented by the discovery of a herbistatic agent via the introduction of a cyclooctatetraene (COT) ring system as a replacement for the benzene ring in metsulfuron methyl [33]. The COT ring system can also exist in its isomeric form as, demonstrated by COT‐MM isomer.

While these examples exhibit an interesting proof of concept for the use of these bioisosteres in the crop protection field, the technical plausibility of using these complex motifs has to be brought into question, as crop protection small molecules cannot command the same prices as prescription pharmaceuticals. This difference, coupled with the economics of these two very different businesses, demonstrates that the cost of production is a key driver when it boils down to the need to select a crop protection candidate for development. It is therefore critical that crop protection chemists consider the cost of goods, as well as the cost per hectare, of their proposed products to ensure that they are a good fit to current and future market needs.

**FIGURE 4:**
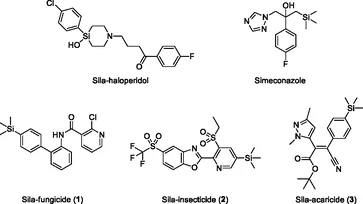
Examples of molecules containing both silicon‐based bioisosteric replacements and where silicon moieties have been introduced to the molecule.

Some of these reasons could partially explain the different approach to bioisosteric replacement optimization that is generally being taken by crop protection chemists. During the conference, Prof. Dr. Maienfisch (CreInSol and Honorary Professor of the East China University of Science and Technology) showcased one key area of his research team's activities on the bioisosteric replacement of carbon with silicon [34, 35]. In many respects the replacement of carbon with silicon is very appropriate, as silicon lies directly below carbon in the periodic table and can therefore be considered a classical switch strategy when optimizing potency, selectivity, and drug‐like properties. Furthermore, silicon is the second most abundant element on Earth and is readily available, making it a cost‐effective replacement for carbon [36]. Many examples of bioactive molecules containing silicon already exist. Notable examples have been reported in both the pharmaceutical and crop protection fields, including the compounds sila‐haloperidol and simeconazole (Figure 4).

Maienfisch and co‐workers have built on these seminal compounds by reporting further research in this field. Examples include the silicon containing fungicide **1** [37], sila‐insecticide **2,** and the sila‐acaricide **3** [38].

**FIGURE 5:**
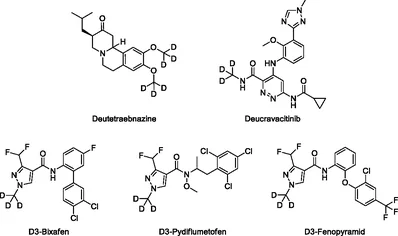
Examples of deuterium‐containing drug and active crop protection molecules.

Another approach that was disclosed in depth during the conference was presented by Prof. Dr. Tracey Pirali (University of Piemonte Orientale, Italy). Her talk focused on the power of replacing hydrogen atoms with the heavy isotope deuterium, an interesting concept used for fine tuning the properties of pharmaceutical candidates, such as metabolism‐mediated toxicity, pharmacokinetic profiles, and circumventing unwanted drug interactions [35, 36]. This strategy has been readily adopted by the medicinal chemistry community, with several drugs already being placed on the market (Figure 5).

Although a plethora of synthetic methods exist, a significant challenge of the deuteration‐replacement strategy is the development of robust chemistry that can allow the targeted replacement of selected hydrogen atoms [39].

However, it is interesting to note that much of the focus in the early years of the “deuterium switch” approach was its use for generating “new” versions of already marketed drugs. This later gave way to mostly concentrating on the tactic for the development of novel drug entities as the patentability of the deuterium‐containing analogs was challenged, particularly regarding the inventive step [40]. It is therefore curious that the “deuterium switch” has started to emerge in the crop protection field in much the same way as it did in pharmaceuticals, as new and generic companies search for intuitive ways to create new products among a background of aging commercial molecules (Figure 5) [41]. This is currently best exemplified by the new wave of Chinese crop protection companies that are presently developing many molecules that are close analogs of already known entities [42].

Bioisosteric replacement strategies have proven time and again that they are powerful tools for the optimization of molecular entities. When comparing how these tactics are deployed in the pharmaceutical and crop protection arenas, it becomes clear that the scale of the different industries is a major driving force for setting the limits of what is possible from the chemical design and cost perspective. In particular, the crop protection industry needs to test the boundaries on this as they design and develop the next generation of commercial products.

### 2.2 Searching New MoA and New Biological Targets in Medicinal and Crop Science

The exploration of biological targets and design of new chemical scaffolds remain crucial to overcome drug and agrodrug resistance, as well as to treat unmet diseases [43, 44, 45, 46, 47, 48, 49, 50, 51]. In this context, natural products (NPs), generally referred to those chemical entities naturally produced by biological organisms (like plants, bacteria, and fungi), have historically played a key role in the discovery of novel active biological compounds. Analysis of approvals between 2014 and 2025 reported by Butler and co‐workers in 2025 [52] and 2026 [53, 54] shows that about 58 NP‐related drugs reached the market, including 45 new chemical entities (NCEs) and 13 NP‐based antibody–drug conjugates (ADCs), while, more broadly, 56 out of 579 globally approved drugs (9.7%) in the 2014–2024 period can be classified as NP‐derived (NP‐D) compounds [52, 53].

**FIGURE 6:**
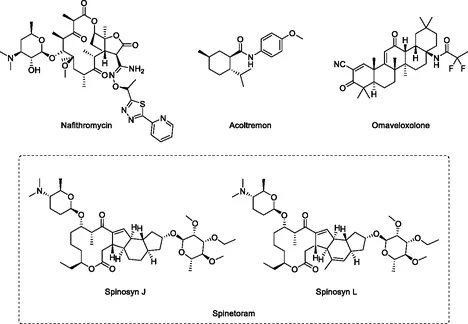
Chemical structure of the NP‐D drugs nafthromycin, acoltremon, and omaveloxolone, and of the NP‐D agrodrug spinetoram.

The general interest in the development of novel therapeutics from natural sources relies on the unique chemical and biological diversity they provide, which represent a source of inspiration for novel pharmacological space. Indeed, by occupying a broader chemical space that remains difficult to replicate using purely synthetic approaches, NPs and NP‐Ds can afford first‐in‐class therapeutics with novel modes of action, as well as active compounds better equipped to combat drug resistance [55, 56, 57, 58, 59, 60, 61]. As an example, in 2025 two NP‐D drugs were approved by U.S. Food and Drug Administration (FDA): the lactone ketolide nafthromycin (WCK 4873) [62], showing a broad spectrum of antibacterial activity, and the (–)‐ menthol derivative acoltremon [63] for dry eye disease (Figure 6). The unique structure of the ketolide nafthromycin, which includes an amidoxime core with a 2‐pyridine‐1,3,4‐thiadiazole chain separated by a rigid four‐atom spacer that has a *cis* double bond, was specifically designed to address the growing issue of resistance to NP‐macrolides like azithromycin, which has become a common first‐line treatment for respiratory infections but has seen diminished effectiveness due to resistant bacterial strains [62, 64]. On the other hand, the approved Alcon's acoltremon (Tryptyr) for dry eye disease is a first‐in‐class agonist of TRPM8, an ion channel that detects cold and menthol and that is involved in tear production. Another valuable example is the 2023 FDA approval of omaveloxolone, a derivative of oleanolic acid easily accessible from multiple plant extracts, which represents the first available treatment for Friedreich ataxia, a severe neurodegenerative disease (Figure 5) [63].

Interestingly, in the last decade, NPs found a new role also in the development of ADCs, which will be further discussed in Section 3. Just to mention, in 2025, the NP‐ADC telisotuzumab vedotin, bearing a dolostatin derivative as bioactive compound (monomethyl auristatin E), was approved for tumor treatment [65], and at least additional 46 NP‐ADCs are currently in Phase II and beyond.

Although these remarkable examples have demonstrated that NPs remain a vital source of new therapeutics and 33 new pharmacophores are currently in development, the limited discovery of truly new pharmacophores in recent years (only one in the past 15 years, represented by the sponge‐derived PM742 patented in 2019 [66]) suggests a worrying decrease of research and investment in this field. This slowdown in NP discovery probably derives from the rise of emerging technologies (i.e., biological drugs) and advancement in the synthesis and development of simpler small molecules. In addition, the average development time for the approved NPs between 2014 and 2025 was 10–15 years from initial discovery to regulatory approval, a timeline that requires a huge amount of investment. Nevertheless, NPs clearly offer chemical and biological diversity that is difficult to replicate with simpler small molecules.

Incontrast, the interest in NPs for crop protection has risen during the past 20 years, affording an increased number of newly identified NP compounds [61, 67]. Currently, about 17% of all crop protection compounds regulated by the EPA and listed within USDA's National Organic Program (NOP) rules are inspired by NPs, a source that still remains one of the most widely used in R&D across industry and academia [61].

Interestingly, natural and NPs‐D have had the largest impact on insecticides compared to herbicides and fungicides, with 31% of the approved insecticides directly related to NPs. The limited contribution of NPs to commercial herbicides may reflect chemical ecology considerations, as plants are generally less likely than bacteria or fungi to produce highly potent phytotoxins. One of the most recent NP‐Ds approved for insecticidal applications is spinetoram, a semisynthetic derivative of the natural insecticide spinosad, which has been commercially available since 1997 as a broad‐spectrum insecticide [68]. Over the years, the structural novelty and potent insecticidal activity of spinosad prompted the investigation of semisynthetic derivatives [68, 69]. In particular, the mixture of 3′‐O‐ethyl‐5,6‐dihydro‐spinosyn J and 3′‐O‐ethyl‐spinosyn L, that was named spinetoram (Figure 6), showed greater potency, earlier onset of activity, and longer duration of control against certain insects when compared with spinosad while retaining broad‐spectrum insect control and low environmental effect on non‐target species [68].

**FIGURE 7:**
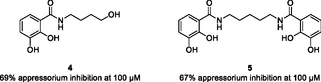
Chemical structure of catechol‐based compounds **4–5** that showed the best in vivo activity for inhibition of spore germination and appressorium formation.

During the congress, Prof. Dr. Michael Christodoulou (University of Milan, Italy) presented an interesting case study [70] on the development of nature‐inspired scaffolds designed to overcome resistance to conventional agrodrugs used against the rice‐blast fungus *Pyricularia oryzae* [71]. More specifically, they investigated for the first time the targeting of ferroptosis in *P. oryzae* by means of siderophores. To better understand the role of siderophores and iron homeostasis in appressoria development and virulence of *P. oryzae*, they prepared a collection of catechol‐containing natural siderophores and various synthetic derivatives. All the derivatives featured a lysine‐ or putrescine‐derived core structure, connected via amide bonds to one or two catechol groups acting as iron‐chelating warheads. This approach led to the development of effective agrodrugs (**4** and **5**) (Figure 7) capable of reducing fungal virulence by interfering with iron availability, a key factor in fungal development and pathogenicity. In particular, catechol‐containing compounds were shown to inhibit appressorium formation, an essential step for host infection, by exploiting their natural ability to chelate iron [70].

Interestingly, this strategy represents an emerging modality also in medicinal chemistry, with studies published in the literature investigating the antivirulence approach of reducing the iron‐mediated virulence and development of bacteria and fungi, for example for the treatment of multidrug‐resistant *Pseudomonas aeruginosa*, *Mycobacterium tuberculosis*, *Mycobacteroides* *abscessus, and Candida albicans* infections [72, 73, 74, 75, 76, 77, 78]. However, no drugs with this mode of action have already reached the market. Therefore, the study of Christodoulou and colleagues represents not only an interesting example of the development of nature‐based compounds in crop protection but also a clear case of cross‐disciplinary inspiration from medicinal chemistry to agrochemical innovation.

Similarly to the investigation of innovative scaffolds with broader chemical space, the identification of new targets remains crucial for the development of new first‐in‐class therapeutics. To date, approximately 500 human proteins are currently targeted by approved drugs [79], but data from the druggable genome suggest that several thousand proteins, ranging from ~3,000 to potentially more than 5,000, could in principle be therapeutically modulated [80], implying that most possible drug targets remain unexplored. In the same way, commercial agrodrugs target a relatively limited set of molecular sites: fewer than ~35 distinct modes of action for herbicides and insecticides each, and a broader set of roughly 95 biochemical targets across all major agrodrug classes (herbicides, fungicides, and insecticides) [81, 82].

**FIGURE 8:**
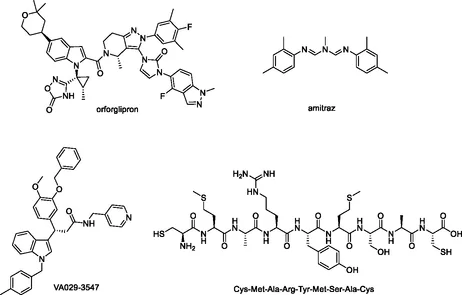
Chemical structures of orforglipron, amitraz, VA029−3547, and CMARYMSAC.

During the conference, Prof. Dr. Necla Birgül Iyison (Boğaziçi University, Turkey) brought our attention on targeting insect's G protein‐coupled receptors (GPCRs) as a promising strategy to induce profound changes in insect physiology [83]. Interestingly, the superfamily of GPCRs is a well‐known target in drug discovery [84, 85], with approximately half of current human medicines being associated with GPCRs or GPCR‐related genes [84]. In the last few years (since 2019), there have been 46 new approvals for drugs that target GPCRs, including seven receptors with first‐time approvals: gastric inhibitory polypeptide (GIP) receptor (GIPR), GPRC5D, C5A1, neurokinin‐3 (NK3), and melanocortin receptor 3, 4, and 5 (MC3/4/5) [84, 86, 87]. The most recent approval was for orforglipron (Foundayo, Eli Lilly) approved to reduce excess body weight and maintain weight reduction long term in adults with obesity or overweight (Figure 8). Orforglipron represents a first‐in‐class, nonpeptide, small‐molecule GLP‐1 receptor agonist designed for oral administration [88, 89].

In comparison, in crop protection the research on the modulation of GPCRs has so far been largely focused on the identification and functional/structural characterization of these receptors using genomic and transcriptomic approaches [90, 91, 92]. There is only one commercially available compound, amitraz (Figure 8), that exerts its function via a GPCR, Octopamine receptor [93]. It is effective against a wide range of phytophagous mites; however, it is also poisonous for animals and humans, which may cause respiratory failure, coma, and hemodynamic instability [93]. In a more recent work, Choi et al. [94] focused attention on the discovery of bioactive small peptides that act by the “receptor interference” (RECEPTORi) mechanism, interfering with the normal binding function of the natural GPCR ligand [94].

In this scenario, Birgül and collaborators conducted integrated in silico and in vitro studies to characterize the structure and function of allatostatin receptor type C (AstR‐C) of pine processionary moth, *T. pityicampa*, a predominant pest residing in Mediterranean countries. In particular, through computational modeling, cell‐based assays, and in vivo evaluations of toxicity and side effects, they identified, for the first time, a set of AlstR‐C ligands with potential as species‐specific insecticides against *T. pityocampa*. The selected agonists (i.e., V029−3547, Figure 8) display strong selectivity toward lepidopteran larvae, while showing negligible impact on coleopteran larvae and adults (*T* *. pit* larvae LD_50_ of 406  and 152 ppm for VO29‐3547 and for AST‐C, the natural ligand of the AstR‐C, respectively), highlighting their suitability for more targeted pest control strategies [95].

### 2.3 Covalent Ligands: A Renaissance Within Modern Drug Discovery

**FIGURE 9:**
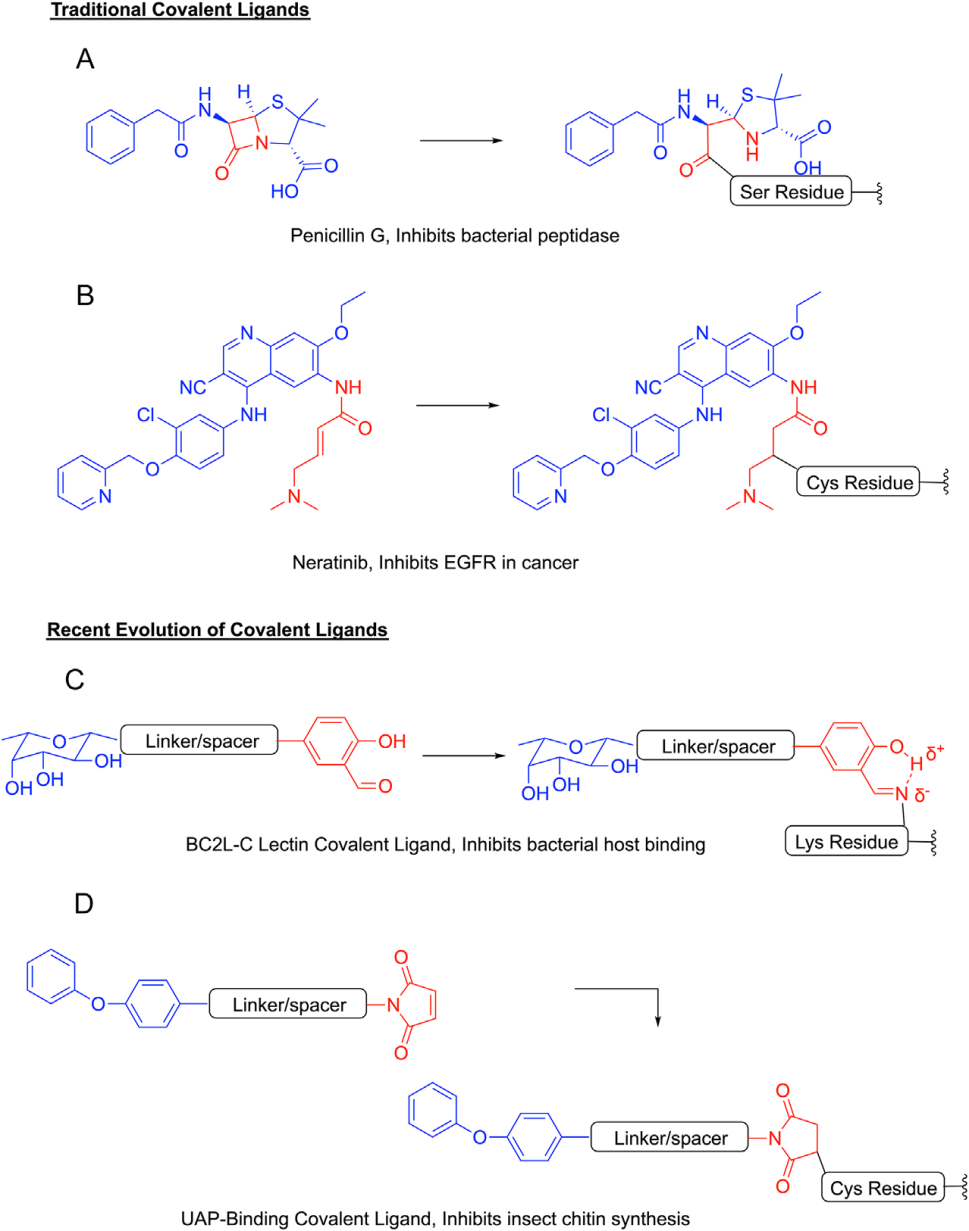
Evolving design of covalent ligands containing moieties that influence noncovalent interaction (blue) and electrophilic covalent bonding (red) using: (A) early, nonreversible, covalent bonding (Penicilin G); (B) reversible covalent bonding (neratinib); (C) (Dr. Mazzotta's BC2L‐C‐lectin‐binding covalent ligand, medicinal chemistry); and (D) (UAP‐binding covalent ligand, agrodrug), reversible covalent bonding with linker/spacer conjugation techniques to modulate orientation of the electrophilic warhead upon interaction of the noncovalent binding moiety.

The covalent modification of proteins has a long history in medicinal chemistry. Great discoveries, while serendipitous, were made in the late 19^th^ and early 20^th^ centuries in the form of acetylsalicylic acid (Aspirin, acetylation of cyclooxygenase) and β‐lactam antibiotics (e.g., penicillin, covalent modification of the vital bacterium DD‐transpeptidase) [96] (Figure 9A); both are great examples of early covalent drugs [97]. However, the serendipitous discoveries of these early covalent drugs betrayed the fact that the mechanism of action, and the reason for their specificity in targets, were something unknown at the time and only elucidated later. These early covalent drugs, such as penicillin, created a false expectation in the ability of covalent drug development, an expectation that hundreds of millions of years of evolution had developed [98, 99]. Subsequent attempts to develop covalent drugs failed to live up to this “evolutionarily computational” drug design. Thus, the field of medicinal chemistry began to filter out the development of covalent drugs for, among other things, the fear of their off‐target effects [100]. However, this downward trend was not to last. Recent FDA‐approved drugs such as azacytidine (inhibiting DNA methyltransferase as an anticancer therapeutic) [101] and Neratinib (developed as an epidermal growth factor receptor inhibitor, for treatment of breast cancer) [102, 103] appear to give credence to the effectiveness of covalent drugs. This is done by utilizing a more specific noncovalent binding moiety as a scaffold, to which there is attached to an electrophilic warhead capable of reversible covalent bonding, designed for specific nucleophilic residues in the protein of interest (Figure 9B) [104]. Thus, with the advent of better computational drug design methods, proteomics, chemistry techniques, etc., the development of covalent drugs, commonly referred to as “covalent ligands,” has found a renaissance within medicinal chemistry.

However, the orientation and proximity of the electrophilic ligand to the protein of interest (POI) is limited by the scaffold of the noncovalent binding vector (as seen in Figure 9A,B). It is with the further application of conjugation techniques to covalent ligands that this renaissance would see additional advancements. Recent advances in medicinal chemistry have moved past the classical covalent drug design strategies (of which we rely on the introduction of an electrophilic warhead which facilitates reversible covalent bonding), with a greater emphasis on the utilization of linkers to conjugate vectors with a drug payload [105, 106]. These targeted strategies can also be used with covalent ligands to decrease their off‐target effect, such as with the use of a vector that noncovalently binds to the protein of interest, bringing the conjugated electrophilic warhead via a linker to the site of reaction. Such linkers have the ability to modulate the length and orientation at which the warhead is in proximity to the nucleophilic amino acid residue of the POI. The work conducted by Dr. Sarah Mazzotta (University of Milan, Italy), as presented at the conference, on covalent ligand drugs for therapeutic use against the infectious *Bukholderia cenocepacia* is one such example. *B. cenocepacia* uses proteins (known as lectins) that bind to host cells via their affinity to sugars, thus providing a target POI for the development of a covalent ligand. In this endeavor, Dr. Mazzotta has utilized linkers to conjugate a sugar with a reversable covalent ligand warhead. Using in silico techniques, a noncovalent ligand association with the target protein was found, containing a L‐frucoside vector for binding the lectin protein (specifically, BC2L‐C‐Nt). This “hit compound” was then redesigned to facilitate covalent bonding to the target protein at a specific lysine residue. The reversible covalent ligand salicylaldehyde was selected as the conjugated warhead. This aldehyde forms a reversible imine bond via a covalent Michael addition reaction with a lysine residue, stabilized by the ortho‐positioned hydroxy group. Various linkers at different lengths were tested to connect the sugar moiety with the electrophilic warhead, allowing for a fine‐tuned interaction of the warhead with the POI that is not limited to the overall scaffold of the ligand or sugar (Figure 9C) [107, 108, 109]. This research was able to produce a conjugated covalent ligand with an IC_50_ at an order of magnitude two times higher than the sugar vector alone, showing the advantages of utilizing conjugation to link effective vectors and electrophilic warheads in reversible covalent ligand development.

With the rise in herbicide and insecticide resistance, there is a need for the development of novel agrochemicals to overcome these challenges in crop protection [110, 111]. Like in medicinal chemistry, covalent modes of action are present in agrochemicals and their use in crop protection. For example, the well‐known covalent agrodrug group is that of the very‐long‐chain fatty acid‐inhibiting (VLCFA) herbicides, whose mode of action is the irreversible covalent bonding to the active‐site cysteine of plant type III polyketide synthase [112, 113]. More recently, the development of an improved insecticide that covalently targets vital enzymes for chitin synthesis—via a conserved cysteine residue—is of particular interest, and was developed using similar techniques as seen with the newfound confidence in developing covalent drugs in medicinal chemistry;, i.e., with the help of conjugation techniques [114]. In this work, a known inhibitor of these chitin synthesis enzymes, *N*‐(4‐nitrophenyl)maleimide (pNPM), was improved by the linkage of a noncovalent binding aromatic moiety (i.e., 4‐phenoxyphenyl group) to the electrophilic warhead component of the original pNPM (Figure 9D). Using initial molecular docking experiments, this linkage and spacing between the vector and warhead greatly increased the position of the maleimide to a targeted nucleophilic cysteine residue of the POI. A flexible alkyl chain was utilized to modulate and optimize the length and spacing of the warhead to the protein, further showcasing the utility of linkers and spacers in optimizing covalent reactions.

Even so, further herbicide development could learn from the use of linkers and spacers to separate the vector from an electrophilic warhead that could help prefer specific nucleophilic amino acid residues conserved in weed species and not crops. In addition, other herbicides with high electrophilic covalent bonding potential could be conjugated to various vectors to bring it to the POI, opening the door to a whole new possibility of targeted covalent herbicide development.

## 3 Emerging Modalities in the Development of Novel Bioactive Compounds

### 3.1 Targeted Protein Degradation (TPD) Strategies

**FIGURE 10:**
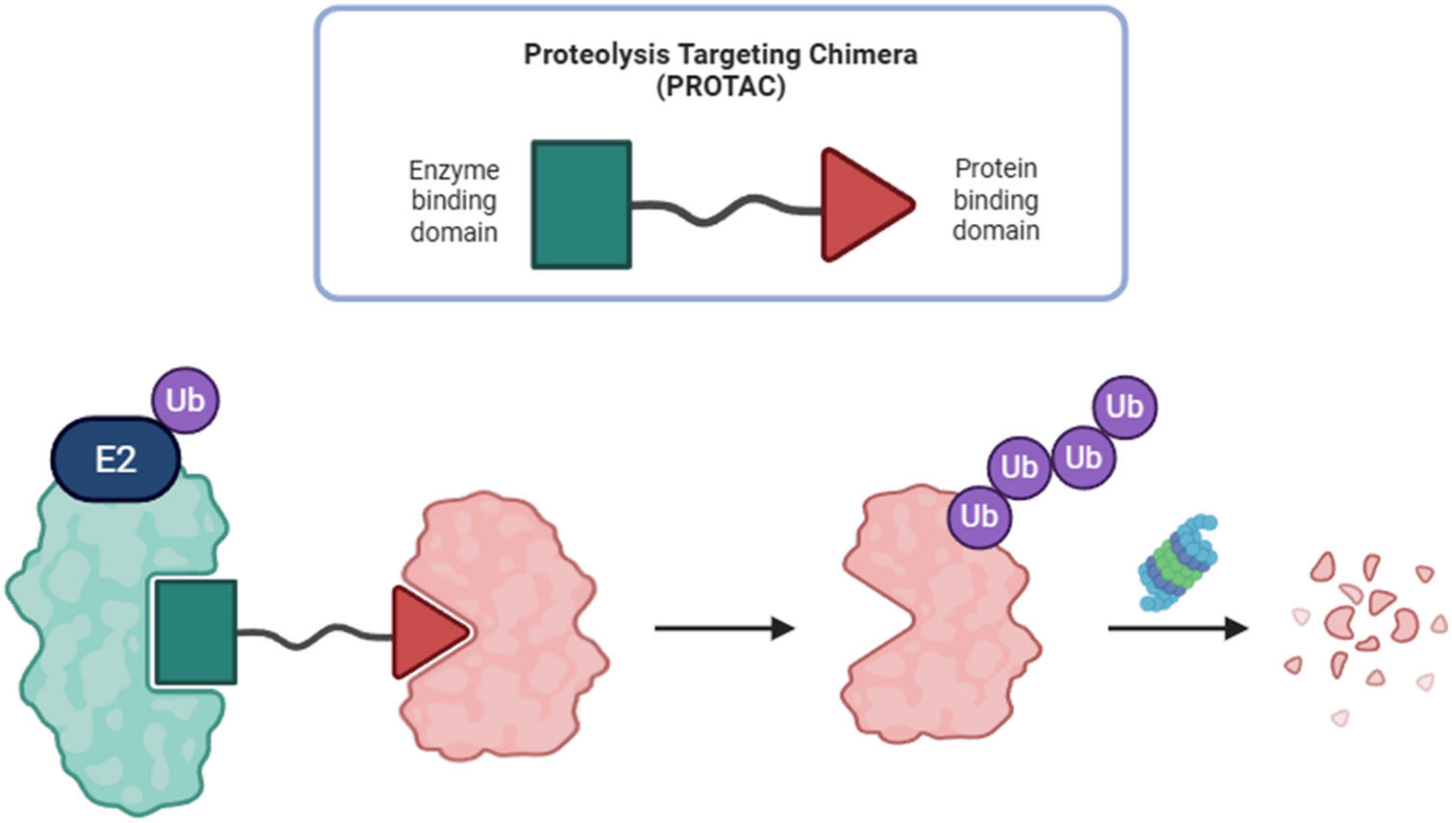
Mechanism of action of PROTACs. PROTACs recruit an E3 ubiquitin ligase to a target protein, promoting its ubiquitination and subsequent degradation by the proteasome in a catalytic manner.

Unlike traditional inhibitors that aim to block protein activity, targeted protein degradation (TPD) strategies employ small molecules to tag the proteins of interest (POIs) for degradation via the ubiquitin‐proteasome system (UPS) or autophagic‐lysosomal system [13, 115, 116]. Among them, PROTAC are synthetic heterobifunctional molecules composed of an E3‐recruiting ligand, a POI‐targeting warhead, and a linker that connects the two [117]. By promoting the formation of a ternary complex comprising the POI, PROTAC, and E3 ligase, these compounds enhance the ubiquitination of the POI, leading to its degradation via the UPS (Figure 10) [13, 118].

**FIGURE 11:**
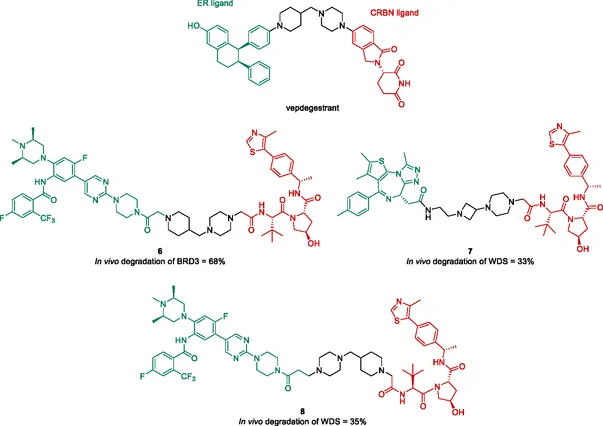
Chemical structures of the first FDA‐ approved PROTAC, vepdegestran, and of the most promising PROTAC for crop protection applications reported by Fourches and collaborators [119] E3 ligase ligands are highlighted in green, POI ligands in red.

Currently, over 40 PROTAC drug candidates are actively being evaluated in clinical trials. Current research primarily focuses on malignancies, targeting well‐characterized proteins such as androgen receptor (AR), Bruton's tyrosine kinase (BTK), interleukin‐1 receptor‐associated kinase 4 (IRAK4), epidermal growth factor receptor (EGFR), and estrogen receptor (ER) (Figure 11) [120, 121].

During the congress, the deep interest in the development of PROTACs as new therapeutics was extensively discussed through the contributions of Dr. Andreas Blum (Merck KgaA, Darmstadt, Germany) and Dr. Jokin Carrillo Arregui (CIMA Universidad de Navarra, Spain). Pharmaceutical companies are indeed attracted by the possibility to target “undruggable” proteins. However, early clinical trials highlight how the intrinsic physicochemical properties of PROTACs systems remain an obstacle to their translation into therapies. Their high molecular weight and polarity often compromise membrane permeability and oral bioavailability, creating significant pharmacokinetic limitations [122, 123]. In addition, their mechanism of action adds further complexity, since effective degradation depends on the formation of a productive ternary complex between the PROTAC, the target protein, and the E3 ligase [124, 125]. Safety is another concern, particularly the risk of off‐target degradation. In CRBN‐based PROTACs, for example, the substrate plasticity of the ligase may cause unintended degradation of proteins such as SALL4 and GSPT1, with potentially unpredictable toxicities [126, 127]. Finally, mutations or loss of components of the E3 ligase complex can drive resistance by impairing ubiquitination and target degradation [128].

Nevertheless, in May 2026, the FDA approved the first PROTAC, Veppanu (vepdegestrant), for patients with ESR1‐mutated, ER+/HER2‐ advanced, or metastatic breast cancers [87]. This orally bioavailable PROTAC induces ER degradation by recruiting the E3 ligase cereblon (CRBN). It as a heterobifunctional molecule consists of lasofoxifene‐based ER binding moiety, lenalidomide‐based E3 ligase CRBN binding moiety, and a rigid N‐containing heterocycle linker (Figure 10) [129, 130, 131]. This approval definitely represents a major milestone in the development of novel PROTACs, demonstrating the real applicability of such systems.

Interestingly, in recent years, the PROTAC concept has also begun to attract attention in the field of crop protection, due to its potentiality to enlarge the number of addressable biological targets. However, the translation of PROTAC research from the pharmaceutical field to agriculture faces several challenges. These include the limited knowledge of suitable E3 ubiquitin ligases in plants and pests, difficulties in ensuring stability and bioavailability under variable environmental conditions, and the need for species‐specific targeting across different biological kingdoms. In addition, large‐scale production must be cost‐effective for field applications, and regulatory requirements, focused on environmental safety and nontarget effects, add further complexity compared to human therapeutics [119, 131].

However, in 2025, Denis Fourches and coworkers from Oerth Bio published a pioneering work where they reported the first examples of PROTACs for agriculture applications (the most potent PROTACs, **6–8**, identified by the authors are reported in Figure 11) [119]. They first demonstrated the abilities of the insect VHL (von Hippel‐Lindau) E3 ligase to degrade endogenous proteins by in vitro and in vivo assays in larvae of fall armyworm (*S. frugiperda*). However, the two targets, *sf*WDS and *sf*BRD3, considered for that study were solely chosen as proof‐of‐concept and were not necessarily intended as insecticidal targets per se.

On the other hand, the only other two available works on PROTAC in pest control [132, 133] reported the insecticide activity evaluation of the developed PROTACs, but they lack key biochemical evidence of ternary complex formation or confirmation of proteasome‐dependent degradation of the targets.

Although the potential of PROTACs in crop science appears highly promising, significant scientific, technical, and regulatory challenges remain, and considerable work is still required before the first commercial product can be realized.

### 3.2 Ligand‐Targeted Drug Conjugates

The limitations of conventional chemotherapy have driven the search for more targeted treatments that can distinguish cancerous cells from healthy ones [134, 135].

**FIGURE 12:**
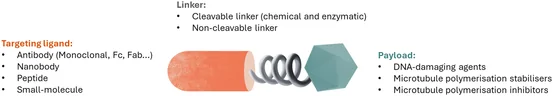
General structure of ligand‐targeted drug conjugates.

Among various modalities, ligand‐targeted drug conjugates (LTDCs) are a promising approach for delivering potent therapeutic agents to cancer cells. They consist of a targeting ligand that recognizes tumor‐associated antigens or receptors, a linker moiety that maintains stability in circulation and enables controlled release at the target site, and a payload, typically a cytotoxic small molecule or radionuclide with antitumor activity (Figure 12) [136, 137].

The most successful example of LTDC is represented by the class of antibody‐drug conjugates (ADCs) [138, 139], in which an antibody‐based antigen‐targeting ligand is combined with cytotoxic small‐molecule compound. The first ADC for tumor treatment became available in 2000 with the FDA approval of gemtuzumab ozogamicin (Mylotarg ) [140], an anti‐CD33 antibody linked via an acid‐labile hydrazone linker to calicheamicin. In the following decade, brentuximab vedotin (Adcetris) [141] and ado‐trastuzumab emtansine (Kadcyla) [142] introduced more stable linkers and potent tubulin inhibitors, representing second‐generation ADCs. Further advancements led to third‐generation conjugates using DNA‐damaging agents such as topoisomerase I inhibitors and PBD dimers. Currently, 19 ADCs have received regulatory approval worldwide, targeting antigens like HER2, CD33, CD30, TROP2, and Nectin‐4 [86, 87, 143].

In parallel to ADCs, other types of targeting units are currently under investigation to overcome several drawbacks correlated to the large size of monoclonal antibody (mAb), such as poor tumor penetration and slow clearance [137, 144].

For instance, smaller version like single‐domain antibodies, also named nanobodies, are one of the latest examples of a targeting unit to incorporate in drug conjugates (sdADCs or NDCs) due to their capability to retain antigen specificity while improving solubility, stability, and tissue penetration thanks to their smaller size compared to mAb [145, 146].

Significant efforts have been also addressed toward the development of synthetic ligands like small peptides or small molecules, that are still able to recognize the targeted receptors. In addition, their lower molecular weight enables rapid tumor penetration, particularly in poorly vascularized tumors [136], and the ease of synthesis allows more sustainable production costs of the respective peptide‐ and small‐molecule drug conjugates (PDCs and SMDCs) [147]. Among the most studied targets, folate receptor‐α (FRα) plays a pivotal role, and the respective folate–drug conjugates have been extensively investigated as a strategy to selectively deliver small‐molecule therapeutics to tumor cells overexpressing the FR [148]. The FRα‐targeted SMDC EC145 (vintafolide), in which the folate moiety is bound to the antimitotic vinca alkaloid DAVLBH through a peptide spacer [149], demonstrated high affinity for FR and advanced to Phase III clinical trials [148, 150]. However, its development was ultimately discontinued after failing to improve progression‐free survival in patients with platinum‐resistant ovarian cancer (NCT01170650) [150]. In contrast, the FR‐targeted imaging agent pafolacianine sodium received FDA approval in 2021 [151], highlighting the continued clinical relevance of FR‐directed strategies. Conjugates bearing carbonic anhydrase IX (CAIX) [152], heat shock protein 90 (HSP90), and prostate‐specific membrane antigen (PSMA) small ligands have also demonstrated encouraging preclinical and early clinical outcomes [153, 154, 155].

Currently, there are no FDA‐approved SMDCs for tumor treatment on the market, although nine are in various stages of clinical trials [156]. One of the most recent examples is represented by VIP236, an SMDC designed by Lerchen and collaborators that is now in Phase I clinical trials for the treatment of metastatic solid tumors by targeting α_v_β_3_ integrins and extracellular cleavage of the 7‐ethyl‐camptothecin payload by neutrophil elastase in the tumor microenvironment [157], proving for the first time the real potential of SMDCs targeting integrin‐overexpressing tumors.

**FIGURE 13:**
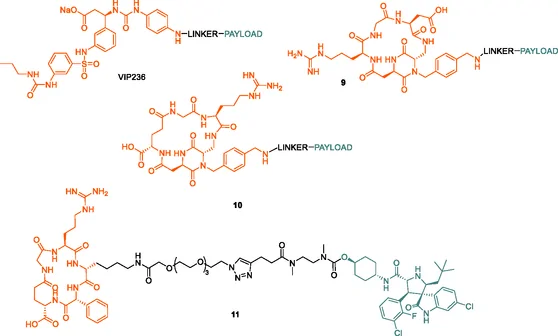
General structure of SMDCs characterized by peptidomimetic α_v_β_3_ integrin ligands. In particular, the SMDCs **9** and **10** are characterized by Cyclo[DKP‐RGD] and Cyclo[DKP‐isoDGR] ligands, respectively. Furthermore, the SMDC **11** represents the first example of dual‐targeting strategy.

Prof. Dr. Umberto Piarulli (University of Insubria, Italy) presented at the congress his research focused on the development of SMDCs able to selectively recognize and deliver potent cytotoxic payloads to α_v_β_3_ and/or α_v_β_5_ integrin‐overexpressing cancer cells. More specifically, Piarulli and co‐workers developed cyclic peptidomimetic ligands incorporating the integrin‐recognizing motifs, Arg‐Gly‐Asp (RGD) or isoAsp‐Gly‐Arg (isoDGR), and a diketopiperazine (DKP) scaffold (Figure 13) [158]. The bifunctional DKP scaffold, acting as a reverse‐turn inducer, is designed to force the peptide chain into a specific conformation, enhancing structural rigidity and stabilizing the molecule, with increased binding affinity for α_v_β_3_ and/or α_v_β_5_ integrin [159].

These small ligands, with low‐nanomolar integrin affinity toward α_v_β_3_ integrin receptor, were successfully inserted into SMDCs bearing either uncleavable or a Cathepsin B‐cleavable linker and cytotoxic payloads like Paclitaxel, α‐amanitine, and dorostatin derivatives (monomethyl auristatin E and F) [160, 161, 162, 163].

Further optimization for improving the poor internalization of these constructs were also shown and included the insertion of cell‐penetrating peptides and the optimization of the drug release strategy with the more efficient Cathepsin B‐cleavable linker Gly‐Pro‐Leu‐Gly developed in their research group [162, 164].

The talk of Professor Piarulli ended with the presentation of a dual‐targeted strategy using α_5_β_1_‐targeting SMDCs. In particular, the restoration of p53 functionality in the tumor environment was demonstrated by employing an MDM2 inhibitor as the payload, combined with a cyclopeptide ligand targeting the α_5_β_1_ integrin receptor (Figure 13), whose overexpression has been shown to correlate with p53 inhibition [165]. Overall, this work demonstrates the versatility of these systems and highlights the promising potential of dual‐targeting methodologies.

AgroDrug conjugates (AgDCs) represent crop science analogs of LTDCs, in which an agrodrug payload is covalently linked to a molecular vector (typically a sugar or an amino acid) to exploit plant membrane transporters, enabling active uptake and systemic distribution [166]. This strategy is attractive because it can enhance biodistribution and target engagement, potentially reducing agrodrug's dosage, limiting environmental contamination, and supporting sustainability goals, although current research remains limited and fragmented [167, 168]. The few studies found in the literature mostly focus on modifying known agrodrugs by conjugating them to sugars or amino acids to improve phloem mobility, with results showing that both the choice of vector and the parent molecule strongly influence transport efficiency (e.g., certain sugar conjugates like β‐D‐fucose or specific amino acids like L‐glutamine enhance mobility more effectively than others) [169, 170, 171].

Despite promising data, the lack of corresponding phloem mobility data for several of these derivatives limits a comprehensive understanding of the relationship between transport and biological activity, emphasizing the need for more integrated and systematic approaches to develop effective AgDCs.

### 3.3 dsRNA‐Based Technologies: Emerging Tools for Targeted Intervention

Double‐stranded RNA (dsRNA) has emerged in medicinal chemistry as a powerful therapeutic agent for the treatment of cancer, metabolic disorders, and viral diseases. Its main applications include dsRNA mimetics that act as immunomodulators and RNA interference (RNAi)‐based therapeutics that enable gene silencing [172].

**FIGURE 14:**
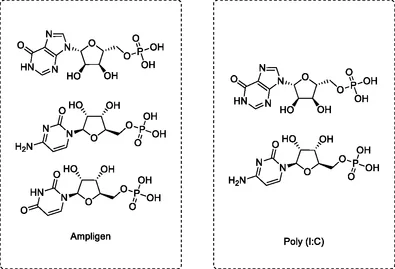
Chemical structures of the immunomodulators poly I:C, and of its more stable derivative, Ampligen.

Among synthetic dsRNA mimetics, the prototypical compound polyinosinic:polycytidylic acid (poly I:C) (Figure 14), first described in 1967, remains extensively used in preclinical models as a potent TLR3 [173] and MDA5 agonist studied in cancer models, including neuroblastoma, where it can induce immune activation and tumor cell death via innate immune signaling pathways [174]. A more stable derivative, rintatolimod (Ampligen) (Figure 14), developed in the 1980s–1990s by AIM ImmunoTech, has progressed into advanced clinical testing and, as of late 2025 to early 2026, is in Phase II–III trials for cancer and antiviral applications, providing one of the most advanced examples of dsRNA‐driven immune activation in humans [175, 176].

On the other hand, since its discovery in 1998 by Andrew Fire and Craig Mello in *Caenorhabditis elegans* (Nobel Prize in Physiology or Medicine in 2006), RNAi [177] has emerged as a conserved and powerful mechanism of post‐transcriptional gene silencing (PTGS) that enables versatile modulation of gene expression [178]. The major forms of RNAi molecules, genome‐derived microRNAs (miRNAs) and exogenous small interfering RNAs (siRNAs), converge into RNA‐induced silencing complexes to regulate target mRNAs at the post‐transcriptional level. Due to its specificity and versatility, RNAi has become a powerful platform for therapeutic development and gene therapy [179].

Currently, 6 siRNA‐based therapeutics have received regulatory approval, highlighting the rapid clinical translation of RNA interference technologies. Patisiran (Figure 14), approved in 2018 as the first siRNA drug, employs lipid nanoparticle (LNP) delivery technology for the treatment of hereditary transthyretin‐mediated (hATTR) amyloidosis [180]. Subsequent approvals include Givosiran (2019) [181], Lumasiran (2020) [182], Inclisiran (2020/2021) [183], Vutrisiran (2022) for hATTR amyloidosis via subcutaneous administration [184], and Nedosiran (2023) for primary hyperoxaluria type 3 [185]. Beyond these approved therapies, numerous siRNA candidates are advancing through Phase III clinical trials and early‐stage development, extending applications beyond rare genetic disorders toward more prevalent diseases, including hypertension and neurodegenerative conditions [186].

As reported by Prof. Dr. Elena Baraldi (University of Bologna, Italy) during the MCPS conference, RNAi approach is particularly relevant also in crop protection given their great potential as a sustainable alternative to traditional agrodrugs. Indeed, RNAi‐based technologies delivered through spray‐induced gene silencing (SIGS) or host‐induced gene silencing (HIGS) offer high specificity and reduced environmental persistence, although their efficacy depends on pathogen biology, environmental conditions, and formulation strategies [187]. Such SIGS strategy was applied by Baraldi and collaborators to deliver dsRNA specifically designed to silence chitin synthase genes for the treatment of gray mold disease on grape and tomato fruits [188]. The external application of these dsRNAs resulted in a significant reduction in the expression level of the target genes, thereby reducing the virulence of *B. Cinerea*. Furthermore, these results further support the use of SIGS‐based strategy for fungal pathogen management [189, 190, 191, 192, 193, 194], by demonstrating that the dsRNA sequences targeting different genes can have the effect of an efficient control of the fungi [188].

One of the earliest notable examples of RNAi‐based applications in crop protection is ledprona, developed by GreenLight Biosciences for the control of the Colorado potato beetle. More recently, the company received an emergency authorization in Belgium for its bioinsecticide Calantha (ledprona), marking the first reported registration of an RNAi‐based crop protection product in the EU [195, 196].

The same company recently announced the U.S. Environmental Protection Agency's (EPA) registration of Norroa (vadescana), the first RNAi‐based treatment specifically designed to combat Varroa Mites, the leading threat to honeybee colonies. This groundbreaking innovation provides beekeepers with a powerful new tool proven to protect these pollinators, which are critical to U.S. agriculture [197].

However, these success examples highlight that, to date, RNAi approaches in insects have mainly targeted juvenile and adult stages, while the egg stage has received comparatively little attention, even though early‐stage intervention could more effectively reduce pest damage.

In this context, Prof. Dr. Francesco Pennacchio (University of Naples, Italy) and colleagues have presented research aimed at developing improved control strategies for *Spodoptera littoralis* by focusing on the silencing of the Sl102 gene, wherein previous studies suggested its role in regulating embryonic development in Lepidoptera. Pennacchio's findings show that Sl102 is effectively expressed throughout the embryonic development of *S. littoralis*, and that its RNAi‐mediated suppression leads to impaired embryogenesis, highlighting its potential as a target for early‐stage pest control [198].

In parallel, new RNAi‐based fungicidal solutions are emerging, such as erysichrona, an officially approved ISO common name for an RNAi fungicide targeting *Erysiphe necator* [199].

Expanding beyond insect and fungal targets, RNAi‐based antiviral strategies are also progressing. In early 2026, tomovircona, developed by Silicon Gene Technologies Co., Ltd, reached a significant regulatory milestone in China as the world's first RNA‐based agrodrug designed to control tobacco mosaic virus [200].

Overall, dsRNA represents a unifying molecular strategy across disciplines, covering both medicinal chemistry and crop science. Recent advances and emerging RNA‐based products highlight its potential either to modulate gene expression and protect plants or to harness immune activation and improve diagnostics and treatments in human health.

### 3.4 Peptides: Versatile Scaffolds for Therapeutic and Agrodrug Innovation

**FIGURE 15:**
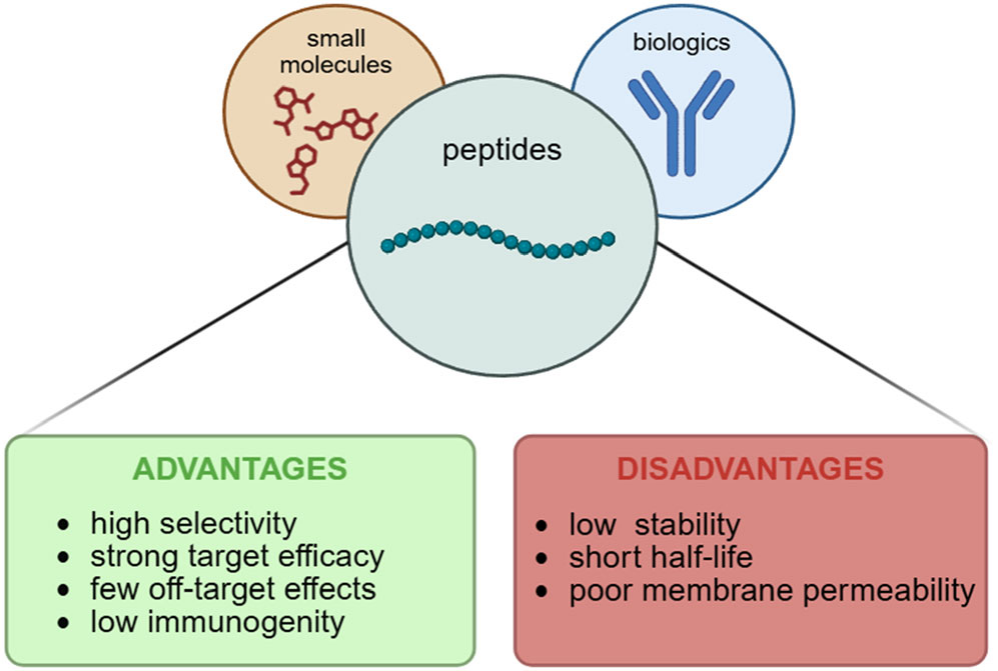
Main advantages and disadvantages of peptide‐based drugs and agrodurgs compared to small molecules and biologics.

The field of peptide therapeutics is steadily expanding in drug discovery and development, addressing a wide range of diseases, such as metabolic disorders, cancer, cardiovascular, immunological, and rare diseases [201, 202, 203]. Over the past decade, peptides have accounted for approximately 8% of FDA‐approved drugs [204]. They provide a unique class of drug candidates, bridging the gap between small molecules and biologics (Figure 15). Their appeal lies in the combination of high binding affinity and remarkable target specificity, together with the ability to access both intra‐ and extracellular targets that are often difficult to modulate using traditional drug modalities [205, 206, 207, 208, 209]. Moreover, peptides retain key recognition features of larger proteins while being smaller, easier to synthesize, and readily tunable to optimize their pharmacological properties. Their relatively larger interaction surface also makes them particularly attractive for targeting protein–protein interactions and addressing challenges such as drug resistance [210, 211, 212]. Despite these advantages, there are challenges in developing peptides as therapeutic agents, including the potential for poor enzymatic stability, low cell‐membrane permeability and bioavailability, rapid clearance, delivery limitations, and high development costs [213, 214, 215]. One of the most successful examples of natural‐deriving peptides that emerged as a class of therapeutics in the management of type 2 diabetes mellitus (T2 DM) and obesity is represented by the class of GLP‐1 (glucagon‐like peptide‐1) receptor agonists [216, 217]. After the first GLP‐1 agonist discovered in 1984, various GLP‐1 analogs have been designed to improve some features such as their half‐life; among them, semaglutide received FDA approval in 2017 for diabetes, which was expanded in 2021 to weight loss and in 2024 to cardiovascular risk reduction in patients overweight or with obesity [218].

Apart of this specific class, approximately 100 peptide drugs are currently in use in the global market, and there are hundreds of new peptides undergoing clinical development and preclinical studies. They span a wide range of therapeutic applications and are integral components of advanced modalities such as ADCs and peptide‐drug conjugates (PDCs) [206]. Peptides have also demonstrated significant clinical relevance in cardiovascular disease, with previously FDA‐approved examples including eptifibatide (Integrilin), bivalirudin (Angiomax), nesiritide (Natrecor), and icatibant (Firazyr). In 2025, the FDA approved the first‐in‐class peptide elamipretide (Forzinity), a synthetic tetrapeptide (D‐Arg‐Dmt‐Lys‐Phe‐NH_2_) containing the unusual amino acid (*S*)‐2‐amino‐3‐(4‐hydroxy‐2,6‐dimethylphenyl) propanoic acid, specifically targeting mitochondria [219, 220]. Notably, natural‐deriving peptides (leucinostatin derivatives) are also emerging as antiprotozoal agents (i.e., Lefleuganan is a clinical stage drug candidate to treat cutaneous leishmaniasis) through the destabilization of the inner mitochondrial membrane of these parasites, which represents a unique MoA that seems to not easily generate drug‐resistant strains [221, 222].

In recent years, peptides have emerged as a rising new star in the field of plant protection. They have been applied as antimicrobials and immune inducers, plant growth regulators, insecticides, and herbicides to protect plants from bacteria, viruses, pests, and weeds. To date, 18 peptides have been commercialized for plant protection [223, 224]. Many of the first peptide‐based crop protection agents were inspired by natural peptides found in biological systems such as microbial metabolites, plant defense peptides, or venom toxins from arthropods, which evolved to interact with insect or microbial targets with remarkable specificity and potency [208]. Interestingly, the last approved (2024) peptide agrodrug, Basim,is a second‐generation peptide based on the chemical structure of the first approved peptide agrodrug, Spear, a derivative of a spider venom neuropeptide that appeared on the market in 2018.

However, the peptides' intrinsic limitations, particularly poor stability and membrane permeability, have driven extensive research aimed at optimizing these natural scaffolds for enabling their practical use in sustainable crop protection [208]. For example, Prof. Dr. Marta De Zotti (University of Padua, Italy) has shown at the congress that structural modifications of the naturally occurring lipopeptaibol trichogin GA IV (1‐octanoyl‐Aib‐Gly‐Leu‐Aib‐Gly–Gly‐Leu‐Aib‐Gly‐Ile‐Lol), produced by the *fungus Trichoderma longibrachiatum*, can significantly enhance antimicrobial activity against plant pathogens by increasing water solubility and membrane interactions through targeted amino acid substitutions. In particular, the most effective peptides, 1‐octanoyl‐Aib‐Lys‐Leu‐Aib‐Lys–Lys‐Leu‐Aib‐Gly‐Ile‐Leu‐NH_2_ (**12**), 1‐octanoyl‐Aib‐Lys‐Leu‐Aib‐Lys‐Gly‐Leu‐Aib‐Lys‐Ile‐Lol (**13**), 1‐octanoyl‐Aib‐Lys‐Leu‐Aib‐Lys‐Gly‐Leu‐Aib‐Lys‐Ile‐Leu‐NH_2_ (**14**), all containing three lysine residues instead of glycine, were able to significantly reduce bacterial titers and symptom development in infected tomato plants with *P. syringae* [225, 226].

Similarly, work presented by Dr. Aurelian Bigot (Syngenta) highlights strategies to overcome delivery limitations of venom‐derived insecticidal peptides. In particular, combining animal neurotoxins with plant‐derived cyclotides such as kalata B1 and B2 can enhance oral toxicity in lepidopteran pests, likely by disrupting the insect midgut barrier and facilitating toxin translocation into the hemocoel [227].

Increasing attention has recently been directed toward micropeptides (miPEPs), small peptides derived from noncoding RNAs (ncRNAs) that are encoded by small open reading frames (sORFs) with a size of less than 100 amino acids [228, 229]. At the MCPS conference, Dr. Jean‐Philippe Combier (CNRS – Le Center national de la recherche scientifique, France) highlighted the role of miPEPs as regulatory molecules capable of modulating plant gene expression and stress responses. In particular, these peptides can enhance the expression of their corresponding microRNAs and thereby influence physiological processes involved in plant growth, development, and resistance to biotic or abiotic stresses [230, 231, 232]. This discovery suggests that miPEPs may represent a promising new class of bioinspired molecules for sustainable crop management, potentially contributing to improved plant resilience while reducing reliance on conventional agrodrugs. To date, no miPEP‐based pesticides are commercially available. However, MPD‐01, a peptide‐based biofungicide developed by Micropep Technologies for the control of fungal diseases such as Asian soybean rust, mildew, and late blight, has recently received a “biochemical‐like active ingredient” classification from the U.S. EPA, marking an important first regulatory milestone toward commercialization [224].

In the pharmaceutical field, miPEPs are also emerging as important regulators of physiological processes such as metabolism, muscle activity, and immune responses. For example, miPEPs like MOTS‐c, PINT87aa, and KRASIM showed good potential in treating cancer and metabolic diseases; however, most of them are still in early experimental stages [233, 234], highlighting the unusual case in which an emerging molecular approach appears to be more advanced in crop protection than in the pharmaceutical field.

### 3.5 Machine Learning and Artificial Intelligence Transforming Small‐Molecule Discovery

The traditional drug discovery paradigm, while successful in delivering thousands of approved medicines, faces mounting pressures from increasing research and development costs, declining productivity, and stringent regulatory requirements. In this context, artificial intelligence (AI) is increasingly recognized as a complementary approach in small‐molecule drug discovery, augmenting traditional methodologies rather than replacing them. By enhancing human expertise and established computational chemistry frameworks, AI offers the potential to mitigate the high costs and prolonged timelines associated with conventional drug development processes [235].

This growing recognition is also reflected at the regulatory level, as highlighted by the 2025 draft guidance from the FDA, “Considerations for the Use of Artificial Intelligence to Support Regulatory Decision Making for Drug and Biological Products” [236].

Across the drug discovery pipeline, AI facilitates target identification through natural language processing and graph‐based integration of multiomics and literature‐derived data; improves hit identification and virtual screening via advanced scoring functions, deep‐learning models, and generative approaches such as diffusion‐based molecular design, and accelerates lead optimization through multiobjective optimization and reinforcement learning strategies that iteratively refine molecular properties [237].

**FIGURE 16:**
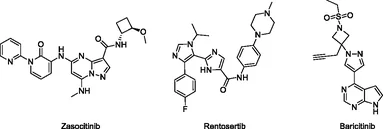
Chemical structures of zasocitinib, rentosertib, and baricitinib.

AI‐driven molecular discovery combined with traditional medicinal chemistry has demonstrated the ability to accelerate specific aspects of drug development, with most clinically relevant compounds emerging after 2018 (Figure 16).

For instance, DSP‐1181, developed by Exscientia in collaboration with Sumitomo Pharma, was designed in approximately 12 months and entered Phase I clinical trials in 2020, representing one of the earliest demonstrations of accelerated AI‐enabled drug discovery. Similarly, rentosertib (ISM001−055), developed by Insilico Medicine, was identified and advanced to clinical testing within ~18–30 months, with Phase I initiated in 2021 and subsequent progression to Phase IIa by 2023–2024 [238].

More advanced candidates, such as zasocitinib (TAK‐279), currently developed by Takeda Pharmaceutical Company, originated from computational design efforts in the late 2010s and have progressed to late‐stage clinical trials over a ~6–8‐year timeline, which is still faster than traditional drug discovery pipelines [239].

AI is increasingly recognized as a promising tool in crop protection too, although its adoption has progressed more slowly than in pharmaceutical applications of computational drug discovery. To help bridge this gap, platforms such as Pesticide Discovery Artificial Intelligence (PDAI) have been developed to tailor AI‐driven molecular design specifically to agrochemical applications. PDAI streamlines the discovery pipeline from target identification to candidate generation, thereby making advanced computational approaches more accessible to non‐specialists. By lowering technical barriers and improving usability, such systems aim to accelerate innovation, reduce development costs, and promote broader interdisciplinary participation in agrodrug research [240].

Within the crop protection industry, it is widely acknowledged that companies are integrating AI technologies into their research and development programs, whether it be internally, via strategic partnerships, or a combination of both. The fast‐paced nature of AI technology developments also gives an opportunity for startups to establish a footprint in the industry. A notable example is provided by Agrematch Ltd, which demonstrated the potential of AI‐driven discovery through the identification and development of the bioherbicide AS9057 in less than 12 months using its AI4AI platform. The compound showed strong postemergence activity against major weed species, including herbicide‐resistant genera such as *Amaranthus* and *Chenopodium*, and is believed to act through a novel mode of action. Early evaluations indicate that AS9057 is effective both as a standalone treatment and in combination with existing herbicides, highlighting its potential relevance for resistance management and sustainable crop protection [241, 242].

At the congress, Dr. Sebastian Klie from Targenomix highlighted how machine learning can integrate public plant datasets (e.g., structural, omics, and annotation data) with proprietary screening and assay results to accelerate early discovery cycles, improve translation to plant efficacy, and generate multiple starting chemotypes with plant‐relevant activity. He emphasized that overcoming resistance and delivering differentiated crop protection solutions requires a shift from phenotype‐first to target‐first discovery strategies. In contrast, a target‐first approach begins with the identification and validation of a specific biological target, such as an essential protein or pathway unique to the pest, pathogen, or weed, prior to chemical design. This strategy enables the rational development of molecules that selectively interact with the intended target while minimizing off‐target effects on crops and non‐target organisms. Consequently, safety and environmental considerations can be incorporated early in the discovery process (“safety‐by‐design”), improving selectivity and reducing ecological impact.

These principles have subsequently been integrated into initiatives such as Bayer‘s “CropKey“ approach to R&D [243].

Looking forward, increased collaboration between academia and industry, particularly through the sharing of data, protocols, code, and models, will be essential to align exploratory and applied research efforts. In addition, emerging areas such as uncertainty quantification and explainable artificial intelligence (XAI) are expected to play a critical role in enhancing model interpretability, reliability, and decision‐making in molecular machine learning applications.

## 4 Conclusion and Outlook

In many respects, the medicinal chemistry and crop protection industries standalone from one another, but they are also united in several aspects of how they conduct their research and development activities and how they both aim to provide for and benefit society. Of all the challenges faced by these two industries, perhaps no obstacle is larger than the one posed by climate change and the way it is altering the nature and distribution of diseases and pests across the globe. Beyond the direct health impacts of climate change, such as exposure to rising temperatures, heatwaves, and extreme weather events, climate change is driving a broader shift in the distribution of infectious diseases, as many pathogens, vectors, and reservoirs are directly or indirectly influenced by climatic conditions. The changing environmental landscape is already shifting patterns of infectious disease burden, with projections indicating that these impacts will further intensify in the future [244, 245, 246]. As a result, the World Bank predicts that an additional 250,000 deaths per year, primarily caused by starvation, malaria, and diarrheal diseases [247], will occur as a consequence of climate change [248].

Crop pathogens, particularly fungi and oomycetes, also exhibit climate‐driven responses, as discussed in‐depth during the congress by Prof. Dr. Daniel Bebber (University of Exeter, UK) [249, 250, 251, 252]. Observational studies identify temperature as the dominant driver of disease prevalence, with soilborne pathogens increasing in relative abundance when exposed to warmer environments. Furthermore, varying precipitation patterns and the increase in atmospheric levels of CO_2_ directly influence the abundance, distribution, and impacts of insect pests and microbial pathogens, while land use intensification and globalized trade accelerate their spread. Additionally, drought and water stress further exacerbate damage, as weakened host plants and reduced natural enemy activity create favorable conditions for pest feeding, though prolonged severe drought or extreme heat can suppress some populations. Conversely, excess rainfall can wash away small insects but often prolongs host plant availability and reduces climatic stress, favoring pest persistence. As you can see, there has maybe never been a more critical time for these industries to act to address the concerns raised by climate change. During these times, we believe that understanding the intrinsic nature of the medicinal chemistry and crop protection industries can mutually benefit their respective research and development pipelines, as well as benefitting society as a whole. The approach of strengthening the interaction between these industries, along with the scientific talents working in them, broadly aligns with the One Health paradigm. As the challenges previously discussed further develop and possibly accelerate, improving cross‐disciplinary collaboration in these areas would better enable the effective stewardship of bioactive molecules, thus enhancing the outlook for the best balancing human, plant, animal, and environmental health. However, it must be recognized that successfully implemented technologies or emerging areas in one field won’t always be transferable to the other. A unique understanding of the pros and cons of each technology, identifying appropriate product concepts, and comprehending how these technologies can benefit the end user are critical for an effective deployment.

## Disclaimer

The opinions expressed in this publication are the view of the author(s) and do not necessarily reflect the opinions or views of ChemMedChem, the Publisher, ChemistryEurope, or the affiliated editors.

## Funding

This work was supported by Ministero dell'Università e della Ricerca (FISA–2022–00922).

## Conflicts of Interest

CropKey's approach has been developed by Bayer CropScience. Oerth Bio is an agricultural biotechnology company founded in 2019 as a joint venture between Bayer CropScience and the biotech firm Arvinas.

## Data Availability

Data sharing not applicable to this article as no datasets were generated or analyzed during the current study.
